# Functional effects of the *TMEM43 *Ser358Leu mutation in the pathogenesis of arrhythmogenic right ventricular cardiomyopathy

**DOI:** 10.1186/1471-2350-13-21

**Published:** 2012-03-29

**Authors:** Revathi Rajkumar, John C Sembrat, Barbara McDonough, Christine E Seidman, Ferhaan Ahmad

**Affiliations:** 1 UPMC Heart and Vascular Institute, Department of Medicine, University of Pittsburgh, Pittsburgh, PA 15213, USA; 2 Department of Genetics, Harvard Medical School and Howard Hughes Medical Institute, Boston, MA 02115, USA; 3 Department of Human Genetics, University of Pittsburgh, Pittsburgh, PA 15213, USA; 4 UPMC Heart and Vascular Institute, University of Pittsburgh, 200 Lothrop Street, Scaife Hall Suite S-558, Mail Stop HPU 01 05 05, Pittsburgh, PA 15213-2582, USA

## Abstract

**Background:**

The Ser358Leu mutation in *TMEM43*, encoding an inner nuclear membrane protein, has been implicated in arrhythmogenic right ventricular cardiomyopathy (ARVC). The pathogenetic mechanisms of this mutation are poorly understood.

**Methods:**

To determine the frequency of *TMEM43 *mutations as a cause of ARVC, we screened 11 ARVC families for mutations in *TMEM43 *and five desmosomal genes previously implicated in the disease. Functional studies were performed in COS-7 cells transfected with wildtype, mutant, and 1:2 wildtype:mutant *TMEM43 *to determine the effect of the Ser358Leu mutation on the stability and cellular localization of TMEM43 and other nuclear envelope and desmosomal proteins, assessed by solubility assays and immunofluorescence imaging. mRNA expression was assessed of genes potentially affected by dysfunction of the nuclear lamina.

**Results:**

Three novel mutations in previously documented desmosomal genes, but no mutations in *TMEM43*, were identified. COS-7 cells transfected with mutant *TMEM43 *exhibited no change in desmosomal stability. Stability and nuclear membrane localization of mutant TMEM43 and of lamin B and emerin were normal. Mutant *TMEM43 *did not alter the expression of genes located on chromosome 13, previously implicated in nuclear envelope protein mutations leading to skeletal muscular dystrophies.

**Conclusions:**

Mutant *TMEM43 *exhibits normal cellular localization and does not disrupt integrity and localization of other nuclear envelope and desmosomal proteins. The pathogenetic role of *TMEM43 *mutations in ARVC remains uncertain.

## Background

Arrhythmogenic right ventricular dysplasia/cardiomyopathy (ARVC) is an inherited disorder characterized by replacement of cardiomyocytes by adipose and fibrous tissue, primarily in the right ventricle (RV). This disruption can result in RV dysfunction, arrhythmias and sudden cardiac death. In the United States, 17% of sudden death victims between the ages of 20 and 40 years had ARVC. A large number of cases are unrecognized because clinical tests are relatively insensitive to *in vivo *detection of functional and structural changes in the RV [1,2]. In approximately 40% of patients, mutations have been identified in genes encoding constituent proteins of cardiac desmosomes, namely, desmocollin-2 (*DSC2*), desmoglein-2 (*DSG2*), desmoplakin (*DSP*), junctional plakoglobin (*JUP*), and plakophilin-2 (*PKP2*)[3]. Mutations can lead to instability of other desmosomal proteins, resulting in their translocation from the cell membrane to the cytoplasm [4,5].

We first mapped by genetic linkage analysis a large family with ARVC to a 9.3 cM region on chromosome 3p23, known as locus ARVC5 [6]. Recently, Merner and colleagues identified a c.1073 C > T mutation, leading to a p.Ser358Leu substitution, in a highly conserved region of transmembrane protein 43 (*TMEM43*) at this locus in the same family [7]. TMEM43, also known as LUMA, is a highly conserved inner nuclear membrane (INM) protein. It has been suggested that TMEM43 can cause pathological changes in the INM by affecting other protein complexes in it [8]. Furthermore, immunostaining of cardiac tissue from subjects with *TMEM43 *mutations indicated reduced expression of TMEM43 and plakoglobin, with TMEM43 showing localization at the sarcolemma [9]. However, little is known about function of TMEM43 and the mechanism by which mutations cause ARVC.

The overall goal of this study was to investigate the mechanisms by which mutations in *TMEM43 *may lead to ARVC. Given the novelty of the *TMEM43 *mutation in the context of previously reported mutations that were primarily in genes encoding desmosomal proteins, we first sought to determine the prevalence of mutations in *TMEM43 *relative to five desmosomal genes, namely, *DSC2, DSG2, DSP, JUP, PKP2*, in 11 ARVC probands. We found three novel mutations in different desmosomal genes, however, *TMEM43 *mutations were absent in these probands. Next, since common mechanisms often underlie each cardiomyopathy even in the setting of genetic heterogeneity, we conducted *in vitro *studies to determine whether the functional abnormalities caused by the rare *TMEM43 *S358L parallel those found in the setting of desmosomal mutations. COS-7 cells were transfected with either wildtype or mutant *TMEM43*, or co-transfected with both. Our studies showed that mutant TMEM43 protein did not disrupt interactions among desmosomal and nuclear envelope proteins, and did not lead to altered cellular localization of itself or of desmosomal and nuclear envelope proteins. Furthermore, mutant *TMEM43 *did not alter the expression of genes as observed in the cardiomyopathy caused by mutations in the nuclear membrane-associated protein, lamin A/C. Therefore, the role of the *TMEM43 *Ser358Leu mutation in ARVC remains uncertain.

## Methods

### Subjects

A total of 11 families with ARVC were recruited by the University of Pittsburgh Cardiovascular Genetics Center and the Harvard Medical School Department of Genetics, in accordance with protocols approved by their respective Institutional Review Boards. Informed consent was obtained from all subjects. Clinical diagnosis of ARVC was based on the recently modified criteria originally proposed by the International Task Force of the European Society of Cardiology and the International Society and Federation of Cardiology [10]. Demographic and clinical data on probands and affected family members in whom a mutation was identified are shown in Table 1.

**Table 1:** Clinical characteristics of ARVC subjects with mutations in desmosomal genes

ARVC diagnostic criteria	Criteria fulfilled	Subject					
		
		Family I.1	Family II.1	Family II.2	Family III.1	Family III.2	Family IV.1
Sex		M	M	M	F	M	M
Age at diagnosis (years)		41	20	17	35	18	16
Global and regional dysfunction and structural alterations	Major	1	1	1		1	1
	Minor				1		
Tissue characterization	Major			1		NA	
	Minor						
Repolarization abnormalities	Major					NA	
	Minor						
Depolarization/conduction abnormalities	Major					NA	
	Minor						
Arrhythmias	Major	1	1	1		NA	1
	Minor						
Family history	Major		1	1	NA	NA	
	Minor						
Mutation		DSG2, c. 208A > G^† ^(Ile70Val), PKP2a, c.397 C > T (Gln133*)	DSC2, C.1234-35insA^†^,(p.Thr412Asnfs*2)	DSC2, C.1234-35insA^†^, (p.Thr412Asnfs*2)	PKP2, c.2540 delT^†^, (p.Leu847Argfs*83)	PKP2, c.2540 delT^†^, (p.Leu847Argfs*83)	PKP2, C.2197-2202delinsG, (p.His733Alafs*8)
Predicted functional effect		DSG2, Ile70Val, Benign					
Mutation screening in 150 normal healthy controls		Absent	Absent	Absent	Absent	Absent	ND (reported earlier)

M, male; F, female; NA, not available; ND, Not determined; †, novel mutations reported in this study; numbering, number of major or minor criteria filled

### Genetic mutation screening

*TMEM43, DSC2, DSG2, DSP, JUP *and *PKP2 *were screened for mutations in all probands, followed by confirmation of any potential mutation in other available family members. Primers were designed using the Primer3 utility http://frodo.wi.mit.edu/. Each identified mutation was screened in 150 unrelated and unaffected control individuals to confirm its absence in normal population. Point mutations were detected by PCR amplification of exons, followed by direct sequencing (ABI). Identified mutations were analyzed for pathogenicity by using (1) protein prediction algorithms PolyPhen http://genetics.bwh.harvard.edu/pph/, SIFT http://sift.jcvi.org/, MUpro http://www.ics.uci.edu/~baldig/mutation.html and PMut http://mmb2.pcb.ub.es:8080/PMut/, (2) evolutionary conservation among species using ClustalW analysis, and (3) localization within a functionally important domain.

### Construction of wildtype and mutant *TMEM43 *expression vectors

#### Wildtype TMEM43

RNA was extracted from normal human heart tissue as described elsewhere [11]. cDNA for *TMEM43 *was synthesized using SuperScript reverse transcriptase (Invitrogen). The sequence was verified by direct sequencing. The resulting fragment was cloned into the pcDNA3.1/NT-GFP-topo eukaryotic expression vector (Invitrogen), which tags the expressed protein with GFP. Cloning and transformation into chemically competent *E. coli *cells was performed according to the manufacturer's instructions. Plasmid DNA was isolated using the QIAprep Spin Miniprep Kit (Qiagen) and quantified on a UV spectrophotometer. The sequence was verified by direct sequencing.

#### Ser358Leu mutant TMEM43

The QuickChange XL Site-Directed Mutagenesis Kit (Stratagene) was used to introduce the Ser358Leu mutation into the wildtype *TMEM43 *expression vector. Mutagenic primers were designed according to the manufacturer's instructions. Plasmid DNA was isolated as described above and presence of the Ser358Leu mutation was verified by direct sequencing.

### Cell culture and transfection

COS-7 cells were cultured in DMEM containing 10% fetal bovine serum (FBS), streptomycin, and penicillin G at concentrations of 100 μg/ml each, and transfected using Lipofectamine 2000 (Invitrogen). Briefly, cells were grown overnight in supplemented DMEM medium and transfected in serum free medium with wildtype plasmid, mutant plasmid, or wildtype and mutant plasmid (at ratios of 1:1 and 1:2) for 6-8 hours. The medium in the plates was then replaced by supplemented DMEM. Cells were harvested five days following transfection.

### Assessment of protein stability by solubility assays

Previous studies have suggested that disruption of cell-cell adhesion because of desmosomal dysfunction is a major mechanism of ARVC. Structural instability of desmosomes leads to localization of constituent proteins in the cytoplasmic instead of the cell membrane fraction following centrifugation in the presence of nondenaturing detergents such as Triton X-100 [5,12]. We assessed the effect of mutant Ser358Leu *TMEM43 *on solubility patterns of four desmosomal proteins (desmocollin-2, desmoglein-2, desmoplakin, and junctional plakoglobin) and the cellular distribution of three nuclear envelope proteins (TMEM43, lamin B and emerin).

#### Solubility of desmosomal proteins

To evaluate the pathogenetic effect of mutant *TMEM43 *on desmosomal proteins, COS 7 cells were transfected with either wildtype, mutant *TMEM43*, or both plasmids at wildtype:mutant ratios of 1:1 and 1:2 for 5 days. Five days following transfection, cells were washed in PBS and lysed in Triton X lysis buffer (20 mM HEPES, pH 7.4, 150 mM NaCl, 0.5 mM CaCl2, and 1% Triton X-100) [4], followed by centrifugation at 20,000 ×*g *for 15 min. The supernatant, comprised of the soluble fraction, and the pellet (insoluble fraction) was resuspended in RIPA buffer (Thermo Scientific). Protease inhibitor tablets (Roche) were added to each of the protein fractions and 40 μg of soluble and insoluble protein fractions were loaded onto a 10% protein gel (Pierce). Immunoblotting was performed as described elsewhere [11]. The following primary antibodies and titers were used following the manufacturer's instructions: desmocollin-2/3 (Zymed Laboratories, #32-600, 1:125), desmoglein-2 (Abcam, #ab14415, 1:1000), desmoplakin (United States Biological, #D3221-89, 1:100), and junctional plakoglobin (Zymed Laboratories, #13-8500, 1:125).

#### Solubility of TMEM43 and other nuclear envelope proteins

Unlike desmosomal and other nuclear envelope proteins, TMEM43 readily solubilizes in Triton X-100. This effect is obviated if the solubility assay is performed on nuclear envelope extract rather than whole cell lysate [8]. Hence, nuclear envelope membranes were extracted from transfected COS- 7 cells, followed by solubility assays. To extract the nuclear envelope, the cytoplasmic fraction and nuclei of transfected cells were separated using ProteoJET cytoplasmic and nuclear protein extraction kit (Fermentas). Nuclei were washed with 20 mM HEPES buffer (pH 7.4) and nuclear envelope membranes were prepared by treatment with heparin and DNase I [13]. Briefly, heparin and DNase I were added to the nuclei suspended in 20 mM HEPES at a final concentration of 0.6 mg/10^8 ^nuclei and 0.5 mg/10^8 ^nuclei, respectively. The nuclei were stirred for 1 hour at 4°C, followed by centrifugation at 12,000 ×*g *for 20 minutes (4°C). The pellet was resuspended in buffer (20 mM HEPES, pH 7.4, 1 mM MgCl_2_, 0.32 M sucrose, 42.8 mM β-mercaptoethanol), and centrifuged again as described above. The pelleted nuclear envelope membranes were lysed with Triton X lysis buffer, followed by separation of the soluble and insoluble fraction, as described earlier. Protease inhibitor tablets (Roche) were added to both fractions. Approximately, 10 μg of soluble and insoluble nuclear envelope protein was loaded on a protein gel and immunoblotting was performed. Differential solubility patterns were assessed for TMEM43 (GFP antibody, Invitrogen, #A11122, 1:1000), lamin B (Santa Cruz, #sc-6217, 1:1000) and emerin (Santa Cruz, #sc-81552, 1:1000) by immunoblotting at the indicated titers.

### Immunofluorescence microscopy

COS-7 cells were grown and transfected in 8-well chamber plates as described above. Cells were fixed using 2% formaldehyde in phosphate buffer saline (PBS), permeabilized with 0.5% Triton-X-100 in PBS, and washed several times with 0.5% bovine serum albumin (BSA) in PBS. Primary antibodies against lamin B (Santa Cruz, #sc-6217, 1:50) and emerin (Santa Cruz, #sc-81552, 1:50) were diluted in 0.5% BSA in PBS. Fixed cells were incubated at 4°C overnight with the antibody at the indicated titer, followed by incubation at room temperature for one hour with appropriate cyanine-3 labeled secondary antibody. Nuclei were stained with Hoechst stain (Invitrogen). Cells were visualized using Nikon Eclipse TS 100 fluorescent microscope.

### Real-time quantitative PCR (QPCR)

Mewborn and colleagues have previously shown that chromosome 13 exhibits a high percentage of misexpressed genes in lamin A mutant hearts. Among these genes, abnormal expression of *KCTD12, LMO7, MBNL2*, and *RAP2A *were shown to be associated with striated muscle dysfunction [14]. Lamin A/C also associates with TMEM43 [8]. Therefore, we quantified the expression of these genes as described [11] in COS-7 cells transfected with either the wildtype or mutant plasmid.

RNA was extracted from cells using Trizol (Invitrogen) and RNA quantity was determined on an Agilent Nanodrop. One μg of total RNA was used to synthesize cDNA using the SuperScript III First-Strand System (Invitrogen). QPCR was performed using Express SYBR GreenER kit with premixed ROX (Invitrogen) on gene specific primers previously published by Mewborn and colleagues [14]. Experiments were performed using two biological replicates and three technical replicates. Samples were run on an ABI Prism 7000 Sequence Detection System. Threshold cycle values were normalized to gylceraldehyde-3-phosphate dehydrogenase. ΔΔCt values were estimated for each gene and unpaired Student's *t*-tests were performed to compare data obtained from wildtype and mutant plasmid transfected cells. A value of *P *< 0.05 was considered significant. Data are expressed as mean ± standard deviation.

## Results

### Clinical evaluation and mutation screening

**Figure 1:**
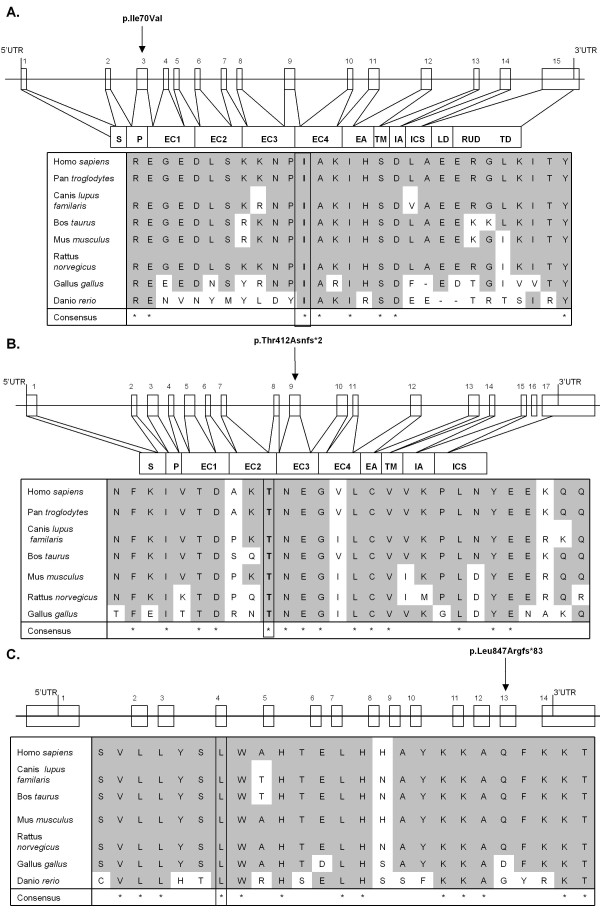
**Significance of novel desmosomal mutations assessed by localization within functionally important domains and their evolutionary conservation**. (A) *DSG2*, p.Ile70Val (c.208A > G); (B) *DSC2*, p.Thr412Asnfs*2 (c.1234_1235insA); (C) *PKP2*, p.L847Rfs*83 (c.2540delT). The numbered white box signifies an exon; the arrow signifies the site of mutation; the grey background indicates identity across species; the box surrounding an amino acid indicates the mutated residue. S, signal domain; P, preprotein domain; EC, extracellular domain; EA, extracellular anchor; TM, transmembrane domain; IA, intracellular anchor domain; ICS, intracellular cadherin-typical segment domain; LD, linker domain; RUD, repeat unit domain; TD, terminal domain.

Novel variants were identified in three desmosomal genes, *DSG2, DSC2 *and *PKP2*, that were absent in 150 control individuals. We also report two known mutations in *PKP2 *(Table 1 and Figure 1).

Family I. The proband (I.1) was a compound heterozygote with a novel *DSG2 *exon 3 mutation, c. 208A > G (p.Ile70Val), and a previously reported *PKP2 *truncation mutation, c.397 C > T (p.Gln133*). The Gln133* mutation has been observed in patients that do not fully satisfy ARVC diagnostic criteria [15]. The proband fulfilled 2 major diagnostic criteria (Table 1) [10]. Although the *DSG2 *Ile70Val mutation was not predicted to be pathological, the Ile residue at this position was highly conserved among species (Figure 1A). Each of two available clinically unaffected family members possessed either the *DSG2 *Ile70Val or the *PKP2 *Gln133* mutation.

Family II. The proband (II.1) had an insertion at c.1234_1235insA in exon 9 of *DSC2 *resulting in a frameshift followed by premature truncation of the protein (p.Thr412Asnfs*2). This region is conserved among species and the predicted protein would lack part of the extracellular, transmembrane domain and cytoplasmic components (Figure 1B). One affected sibling (II.2) shared the same mutation as the proband (Table 1). The clinical manifestation of the disease at a young age in both the proband and the sibling is consistent with the pathogenicity of the *DSC2 *Thr412Asnfs*2 mutation.

Family III. The proband (III.1) had a deletion at c.2540delT in exon 13 of *PKP2*, resulting in a loss of a stop codon and a mutant protein that was 48 amino acids longer than the wildtype protein (p.Leu847Argfs*83). This region is conserved among species (Figure 1C). One other family member shared the same deletion as the proband (III.2). The proband only partially satisfied ARVC diagnostic criteria, probably because the mutant protein functions similarly to the wildtype *PKP2 *protein. There was insufficient clinical information to make a diagnosis in family member III.2.

Family IV. The proband (IV.1) fulfilled the ARVC diagnostic criteria and presented with a previously reported deletion and insertion in exon 11 of *PKP2 *at c.2197-2202delinsG that resulted in a premature truncation of the protein (p.His733Alafs*8) (Table 1) [16].

No mutations in *TMEM43 *were identified in the 11 probands, suggesting that mutations in this gene are a relatively rare cause of ARVC.

### Functional characterization of mutant TMEM43

#### Desmosomal and nuclear envelope protein stability

Previous studies have suggested that disruption of cell-cell adhesion because of desmosomal dysfunction is a major mechanism of ARVC. Structural instability of desmosomes leads to localization of constituent proteins in the cytoplasmic instead of the cell membrane fraction following centrifugation in the presence of nondenaturing detergents [5,12]. We assessed the effect of mutant Ser358Leu *TMEM43 *on solubility patterns of four desmosomal proteins (desmocollin-2, desmoglein-2, desmoplakin, and junctional plakoglobin) and the cellular distribution of three nuclear envelope proteins (TMEM43, lamin B and emerin).

**Figure 2:**
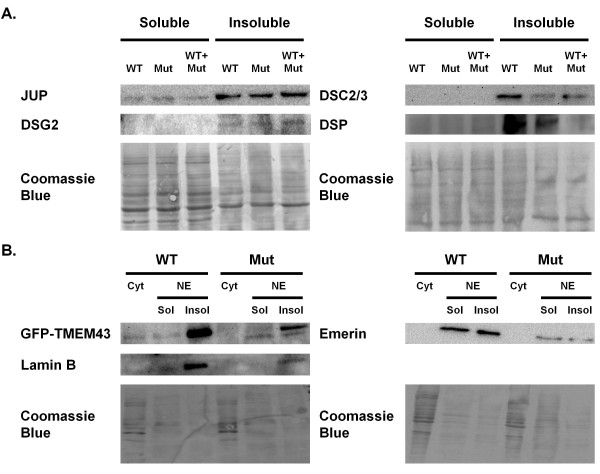
**Solubility assays**. Solubility patterns for (A) desmosomal and (B) nuclear envelope proteins were assessed by immunoblots for soluble and insoluble protein fractions extracted from COS-7 cells transfected with wildtype (WT) *TMEM43*, mutant (Mut) *TMEM43*, or a 1:2 ratio of wildtype to mutant (WT + Mut) *TMEM43*. Sol, soluble fraction containing cytoplasmic (Cyt) proteins; Insol, insoluble fraction containing membrane bound proteins. No differences in solubility patterns were found, suggesting that the presence of mutant *TMEM43 *did not alter the stability of TMEM43 or of other desmosomal or nuclear envelope proteins.

**Figure 3:**
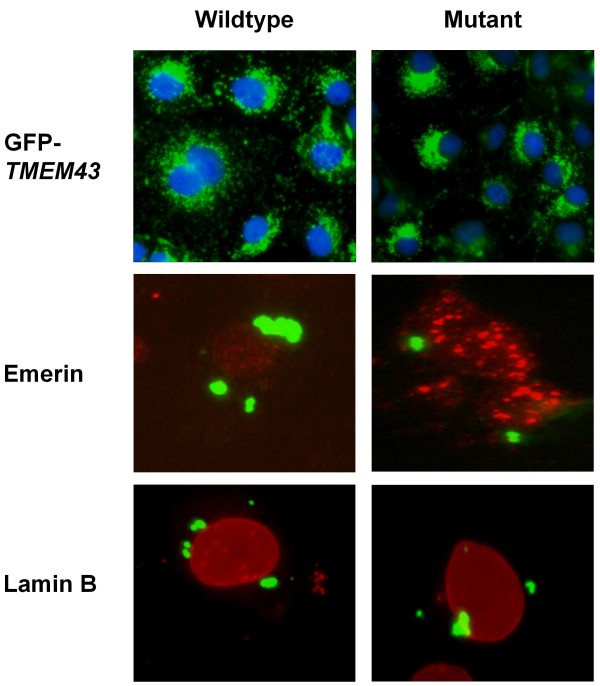
**Immunofluorescence imaging of nuclear envelope proteins**. There was similar distribution of GFP tagged *TMEM43*, emerin and lamin B in cells transfected with wildtype or mutant *TMEM43*. Green color, GFP-labeled TMEM43; red color, emerin and lamin B; blue color, Hoechst stain for nuclei.

Solubility assays indicated similar localization of desmocollin-2, desmoglein-2, desmoplakin, and junctional plakoglobin in the cell membrane bound insoluble fraction, irrespective of the transfection dosage for both wildtype and mutant *TMEM43 *(Figure 2A, data not shown for 1:1 dosage). Mutant TMEM43 and lamin B were present in the insoluble nuclear membrane bound fraction (Figure 2B). Although emerin was present in both soluble and insoluble fractions, there was no evident change in the ratio between the two fractions. Immunofluorescence microscopy also showed that TMEM43, lamin B, and emerin were localized to the nuclear membrane in both wildtype and mutant *TMEM43 *transfected cells (Figure 3). Thus, these data suggest that mutant *TMEM43 *does not change its own localization or that of its binding partners lamin B and emerin.

### Gene expression changes

We examined the effect of mutant *TMEM43 *on mRNA expression of four genes, *LMO7, KCTD12, MBNL2 *and *RAP2A*, located on chromosome 13, that have been shown to have abnormal expression in the setting of laminopathies with striated muscle dysfunction [14]. QPCR preformed on these four genes showed no significant change in their mRNA expression in cells transfected with wildtype and mutant *TMEM43*, respectively (Table 2). Thus, while TMEM43 interacts with lamin A, we conclude that mutant TMEM43 protein, unlike mutant lamin A, does not alter expression of at least four representative genes on chromosome 13.

**Table 2:** mRNA expression in selected genes on chromosome 13 following transfection with either wildtype or mutant *TMEM43 *plasmid

Gene	Fold change (mutant vs. wildtype)	*p*
*KCTD12*	0.73 ± 0.04/1.00 ± 1.07	0.29
*LMO7*	1.11 ± 0.19/1.00 ± 0.03	0.20
*MBNL2*	1.34 ± 0.25/1.00 ± 0.08	0.08
*RAP2A*	1.29 ± 0.44/1.00 ± 0.06	0.18

Data are represented as mean ± standard deviation

## Discussion

The mechanisms by which mutant desmosomal proteins cause ARVC have been partially elucidated. Previous studies have shown that overexpression in cell lines of mutant desmoplakin and desmocollin-2 associated with ARVC results in cytoplasmic distribution of these proteins instead of normal membrane localization [5,12] and knockdown of *DSP *leads to nuclear localization of plakoglobin [17]. In *Pkp*^-/- ^null mice, plakophilin-2 and plakoglobin were found to be essential for adherence of desmoplakin to partner desmosomal proteins [4]. More recently, the Ser358Leu mutation in *TMEM43 *has been suggested [7] as a putative cause for ARVC in a large kindred that we mapped to chromosome 3p23 [6]. Furthermore, decreased expression of plakoglobin has been shown in tissues of patients with ARVC including those with *TMEM43 *mutations [9,18]. To uncover potential mechanisms by which *TMEM43 *mutations may lead to ARVC, we first sought to determine its frequency in the ARVC population by screening for mutations in this gene along with five other desmosomal genes; and we then examined the specific effects of Ser358Leu mutant *TMEM43 *on stability of desmosomal and nuclear envelope proteins and on gene expression in the COS-7 heterologous cells system.

Our patient cohort lacked *TMEM43 *mutations, suggesting that mutations in this gene are a relatively rare cause of ARVC and consistent with a previously reported screen [8]. However, we have identified three novel mutations in three probands. The presence of two mutations, i.e., p.Ile70Val in *DSG2 *and p.Gln133* in *PKP2 *in the Family I proband, contrasted with unaffected family members who had only one mutation each, suggests that co-segregation of these two mutations in the proband may have led to the development of ARVC. Earlier studies have also suggested that digenic and compound heterozygous mutations are frequent in desmosomal genes and may be responsible for severe ARVC [19-21]. The *DSC2 *(p.Thr412Asnfs*2) frameshift mutation is likely pathogenic since it results in a truncated protein and both individuals in Family II with this mutation fulfilled ARVC diagnostic criteria.

As discussed earlier, ARVC is primarily a disease of the desmosomes. Based on this observation, we hypothesized that mutant TMEM43 protein would disrupt structure and function of desmocollin-2, desmoglein-2, desmoplakin, and junctional plakoglobin, leading to ARVC. However, we found no evidence of disruption or mislocalization of mutant TMEM43 or of desmosomal proteins in transfected COS-7 cells. Thus, our data suggest that mutant *TMEM43 *does not directly affect desmosomal protein localization on even increasing the dosage of mutant protein.

TMEM43 is an INM protein that has a large hydrophilic domain located in the endoplasmic reticulum (ER) lumen. It helps to maintain the nuclear envelope structure by organizing protein complexes in the INM and it is known to associate with other nuclear envelope proteins such as lamins and emerin [8]. Lamins have been extensively studied primarily because mutations in lamin A/C lead to dilated cardiomyopathy [22] and lamin B maintains nuclear shape and mechanical integrity [23]. Since mutations in emerin cause Emery-Dreifuss muscular dystrophy and TMEM43 can affect emerin distribution, it has been suggested that *TMEM43 *mutations could result in muscular dystrophy [8]. We therefore sought to determine whether mutant *TMEM43 *affects the localization of emerin and lamin B, which in turn might lead to ARVC. However, our solubility and immunofluorescence experiments indicated no such abnormalities.

As mentioned above, mutations in lamin A can lead to dilated cardiomyopathy. Lamin A/C, encoded by *LMNA*, is also a member of the LINC complex (linker of nucleoskeleton and cytoskeleton) that links the nucleus to the cytoplasm and also associates with TMEM43. Mewborn and colleagues showed that lamin A mutants exhibited misexpression of genes located on chromosome 13, which may potentially lead to laminopathies [14]. We therefore examined the effect of mutant TMEM43 protein on mRNA expression of four genes, namely, *LMO7, KCTD12, MBNL2 *and *RAP2A*, all of which are located on chromosome 13, are associated with striated muscle dysfunction, and exhibit abnormal expression in the presence of a *LMNA *mutation [14]. However, in the presence of mutant *TMEM43*, these genes exhibited normal mRNA expression.

## Conclusions

We identified three new mutations in desmosomal genes in 11 probands screen; however, no *TMEM43 *mutations were identified. The absence of a mutation in majority of our ARVC patients, as in prior studies, suggests the presence of other genetic contributors to this disease. This study also confirms normal cellular localization and stability of the analyzed desmosomal and nuclear proteins in COS 7 cells transfected with mutant *TMEM43*. Furthermore, mutant *TMEM43 *did not alter the expression of genes that are suggested to be associated with laminopathies. A limitation of this study is that a heterologous cell system was used, which may not completely reflect the effect of *TMEM43 *mutations in cardiomyocytes. Additional studies in cardiomyocytes may be required to uncover a clearer pathogenetic link between the Ser358Leu mutation in *TMEM43 *and the development of ARVC.

## Competing interests

The authors declare that they have no competing interests.

## Authors' contributions

RR participated in the conception and design of the study, performed all molecular biology experiments, analyzed and interpreted the data, and drafted the manuscript. JS maintained cell culture, performed nuclear protein isolation and immunoblotting. BM carried out acquisition of clinical data. CES recruited and evaluated subjects at Harvard Medical School. FA conceived the study, participated in its design and coordination, recruited and evaluated subjects from the University of Pittsburgh, assisted with data interpretation, and wrote the final manuscript. All authors read and approved the final manuscript.

## Pre-publication history

The pre-publication history for this paper can be accessed here:

http://www.biomedcentral.com/1471-2350/13/21/prepub
